# The effects of perineal disinfection on infant’s oral microflora after transvaginal examination during delivery

**DOI:** 10.1186/s12884-019-2350-3

**Published:** 2019-06-24

**Authors:** Hongping Li, Shaoyun Chen, Lijuan Wu, Huilin Wang, Kelin Xiao, Yanling Gao, Yao Li, Huiqin Li, Bin Xiao, Yuanfang Zhu

**Affiliations:** 1 Maternal-Fetal Medicine Institute, Bao’an Maternity and Child Health Hospital, Jinan University, 56 Yulv road, Bao’an, Shenzhen, 518100 China; 2 Shenzhen Children’s Hospital, Shenzhen, 518100 China

**Keywords:** Povidone iodine, Perineal disinfection, Infant’s oral microflora, 16S rRNA, Lactobacillus

## Abstract

**Background:**

Early life microflora is an important determinant of immune and metabolic development and may have lasting consequences. However, the mode of delivery and the effect of povidone iodine disinfection on neonatal oral microflora colonization are still unclear. The objective of the study was to understand the effects of the use of polyvidone iodine on infant’s oral microflora after transvaginal examination during delivery, provided data support for the establishment of neonatal oral microflora health.

**Methods:**

A total of 20 cases of full-term neonatal delivered in October 2017 in Shenzhen Bao’an Maternity and Child Health Hospital through vaginal delivery. These neonates were randomly divided into two groups, the conventional disinfection group and the non-disinfection group. Simultaneously, 10 infants with elective cesarean section were taken as comparison. With Illumina MiSeq platform, 16S rRNA V3-V4 sequencing method was used to analyze bacterial DNA of oral secretions.

**Results:**

At the phylum level, compared to the non-disinfection group, higher relative abundance of Bacteroidetes and Proteobacteria, and lower proportion of Firmicutes were observed in the cesarean section group and the disinfection group. As main composition of phylum Firmicutes, genus Lactobacillus presented extremely low in the cesarean section group and the disinfection group, whereas it was the absolute dominant bacteria in the non-disinfection group. Compared with the caesarean section group, only Lactobacillus increased in majority of the non-disinfection group. There was no increase in Lactobacillus in the disinfection group, but Prevotella, Escherichia-Shigella, Staphyloccus, and Klebsiella increased significantly. Through KEGG pathway analysis, we found that there were more harmful pathways such as *staphylococcus aureus* infection, viral myocarditis and sporulation in the disinfection group.

**Conclusions:**

The mode of delivery affects the infant’s Lactobacillus obtained from the mother. Moreover, vulvar disinfection played an important part in the colonization of neonatal oral microbiota. And the impact of the first oral colonizers on infant health needs further follow-up investigations.

## Background

Infancy is an important stage in the formation of human oral microflora, and the programmed establishment of normal oral microflora is crucial for infant’s health and essential to elucidate the early stages of microbiota development [1]. Infantile oral microbiota plays an important role in microbial community and body health, its interactions helps the human body against invasion of undesirable stimulation outside [2, 3]. In addition, dysbiosis of oral microbiome closely related to dental caries, periodonititis and oral mucosal diseases [4–6]. Mode of delivery, feeding type, pregnancy outcomes, antibiotics and so on, these factors also affected the acquisition of the first oral microbial colonizers of neonates [7, 8]. While the colonization process of the infant gut microbiome has been studied extensively [9–11], the colonization of the neonatal oral microbiome is still unclear.

For a host-microbial interaction in utero, and the fetus commonly encounters bacteria that inhabit the maternal vaginal tract after rupture of fetal membranse, status of mother is the primary source of oral bacterial for newborns [12]. However, the latest edition of the medical education book “Obstetrics and Gynecology” in China shows that medical staff need to use povidone iodine to disinfect the vulva before transvaginal examination during maternal delivery [13]. Up to now, almost all hospitals in China operate according to this rule. However, the effects of the medical behavior on the normal colonization of the infant’s oral microflora are not clear. In this paper, we aim to clarify the effects of perineal disinfection on infant’s oral microflora after transvaginal examination during delivery, so we take samples of infant’s oral secretions after the infant’s fetal head has been delivered. With the advancement and maturity of high-throughput sequencing technology, second-generation sequencing technology was used to sequence bacterial 16S rRNA (ribosomal RNA), and the bioinformatics technology was used to analyze the composition of the bacteria.

## Methods

All procedures and experiments in this research were supported by the Shenzhen Bao’an Maternity and Child Health Hospital’s ethics committee (the committee’s reference number: QKTLL-2017-05-04). Collecting samples and sequencing were performed in accordance to relevant guidelines and regulations. Referring to the guidelines of Shenzhen Bao’an Maternity and Child Health Hospital, written informed consents were obtained from the parents of infants.

### Research objective

A total of 30 infants were recruited in this research, including 20 natural deliver and 10 cesarean section neonates in October 2017 in Shenzhen Bao’an Maternity and Child Health Hospital, whose families agreed to be included in this study, were selected as the subject. Among 20 participants in natural childbirth, 10 cases’ vulvas were not sterilized before the transvaginal examination, named as the non-disinfection group. Then accordingly, the remaining 10 cases were examined by routinely disinfected by povidone iodine. Taken together, our study included a total of 3 different groups, namely the disinfection group (D), the non-disinfection group (V) and the cesarean section group (C). Mothers of caesarean section infants chose caesarean section because of “scar uterus”, “social factors” and “head basin asymmetry”.

Inclusion criteria: mothers’ gestational age > 37 weeks, neonates’ birth weight > 2500 g, mothers’ pre-pregnancy body mass index (BMI) 19–25, and mothers and fetuses are in good health.

Exclusion criteria: neonates’ birth weight < 2500 g, developmental deformity, postnatal asphyxia, respiratory instability, mothers with complications during pregnancy or chronic metabolic disease, or intake of antibiotics during the whole pregnancy, dystocia required invasive operators such as episiotomy, midwifery, fetal head suction.

### Samples collection and preservation

Neonates’ oral secretions were immediately collected by medical staff in the operating room through using sterile syringes within 1 min after the baby has been delivered. Then the sterile syringes containing the samples were sealed in sterile bags and stored in an − 80 °C low temperature refrigerator until further processing.

### DNA isolation and pretreatment

Bacterial total DNA was isolated by using PowerSoil® DNA Isolation Kit (QIAGEN, Germany) according to the manufacturer’s protocol. DNA concentrations were measured by NanoDrop ND-1000 Spectrophotometer (NanoDrop Technologies, Wilmington, DE, USA), and DNA quality was evaluated based on the absorbance ratios at 260/280 nm and 260/230 nm, simultaneously. Usually DNA with a 260/280 absorbance ratio in the range of 1.8–2.0 is defined as good quality. Subsequently, bacterial DNA was quantified using Qubit dsDNA HS Assay kit (Life Technologies). The qualified DNA samples were performed to the first round of polymerase chain reaction (PCR) by 16S V3-V4 region primers (341R:CCTACGGGNGGCWGCAG, 806F:GACTACHVGGGTATCTAATCC). The first round of PCR product purification followed by sequencing primers and adapters for second round of PCR. Moreover, the product was purified and quantified after amplification, followed by sequencing using the MiSeq platform.

### 16S rRNA sequencing and data analysis

16S rRNA sequencing was performed using the MiSeq platform in the double-ended sequencing mode. Based on the distribution characteristics of low-quality bases at the end of the sequencing reads, quality was initially evaluated with FastQC. Then de-multiplexing to remove PhiX sequences and reads assignments based on dual-index barcodes were performed using custom Perl scripts. The selected high quality sequences were further processed using Mothur pipeline according to the Mothur SOP [14]. In a first step, the forward and reverse reads were merged into tags. Tags exhibiting any ambiguous positions or containing a more than 8-base homopolymer were subsequently removed. Next, tags were aligned to the SILVA reference database [15], these failed to align to the correct location within 16S rRNA gene were culled. Aligned tags were simplified, dereplicated, and denoised with mother implementation of the Single Linkage Preclustering algorithm [16]. The resulting tags were screened for presence of chimeras using UCHIME [17]. Taxonomic classification was done based on Ribosomal Database Project [18] training set ver.10, followed by no-bacterial sequence removal. Finally, tags were clustered into OTUs (operational taxonomic units) at distance 0.03. In this study, KEGG (Kyoto Encyclopedia of Genes and Genomes) [19] database was used to annotate microbiome genes with PICRUST (Phylogenetic Investigation of Communities by Reconstruction of Unobserved States)program [20].

### Statistical analysis

All experimental data were analyzed and plotted using the R software (version3.3.3). Continuous variables were presented as mean ± standard deviation ($\overline{x}\kern0.5em \pm \kern0.5em s$), and the data of three experimental groups were compared using the ANOVA test. Categorical variables were presented as sample number / total number, and relevant group comparisons were performed using Chi-square test. *P* value of 0.05 or less was considered statistically significant, and test level α = 0.05.

Taxa with differentiating relative abundance were identified using LDA Effect Size (LEfSe: Linear Discriminant Analysis Effect Size) program [21]. The threshold for the logarithmic LDA score was set at 2.0 for biomarker discovery.

The STAMP program [22] was used in this study to generate an extended error bar plot to show the properties which differed significantly between two groups with filter parameters (*q* value < 0.05).

## Results

### Comparison and analysis of clinical characteristics of mothers and infants

A total of 30 infants were recruited in this research, including 20 natural deliver and 10 cesarean section neonates. Among 20 participants in natural childbirth, 10 cases’ vulvas were not sterilized before the transvaginal examination, named as the non-disinfection group. Then accordingly, the remaining 10 cases were examined by routinely disinfected by povidone iodine. Taken together, our study included a total of 3 different groups, namely the disinfection group (D), the non-disinfection group (V) and the cesarean section group (C). In order to study the effects of the single variable (povidone iodine disinfection) on the infant’s oral microflora, the interference of other variables must be excluded. From Table 1, it can be seen that the *P* values were greater than 0.05 in both categorical variables and continuous variables through statistical analysis. Therefore, the differences were not statistically significant in mother’s age, infant sex, gestational week, birth weight, gestational weight gain, and gestational diabetes mellitus among the three groups, that is, the effects of the differences in these variables on the results was negligible.

**Table 1:** Clinical characteristic of mothers and infants from different groups (disinfection group, non-disinfection group and cesarean section group)

Characteristics	D(*N* = 10)	V(*N* = 10)	C (*N* = 10)	D*vs*V*vs*C *P* value
Mother’s age	28.5 ± 3.5	29.3 ± 3.5	27.3 ± 3.8	0.46
Infant sex (F/M)	5/5	5/5	6/4	0.87
Gestational week	39.5 ± 1.4	39.4 ± 0.7	39.7 ± 1.2	0.87
Birth weight (g)	3130 ± 393	3114 ± 293	3450 ± 451	0.11
Gestational weight gain (kg)	13.2 ± 5.2	13.1 ± 3.2	14 ± 3.8	0.87
Gestational diabetes mellitus	0	1	1	0.59

D represents the disinfection group, V represents the non-disinfection group and C represents the cesarean section group. Three experimental groups were compared using the ANOVA test. Categorical variables were presented as sample number / total number, and relevant group comparisons were performed using Chi-square test. *P* value of 0.05 or less was considered statistically significant, and test level α = 0.05

### Difference on oral microbial diversity in response to disinfection

**Fig. 1:**
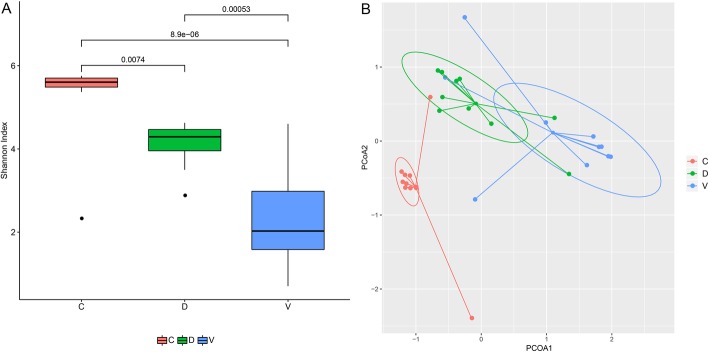
Pairwise comparisons of the microbiome diversity of the three groups. D represents the disinfection group, V represents the non-disinfection group and C represents the cesarean section group. **a** The Shannon index was shown as an estimator. **b** PCoA plot based on the taxa abundance

Average value of Shannon index was 5.27 ± 1.04, 4.11 ± 0.55, and 2.26 ± 1.15 for C, D, and V groups respectively. Further statistical analysis showed that the V group had the smallest microbial diversity among the three groups, both the C (*p* < 0.01) and D *(p* < 0.01) groups with significantly higher value than the V group. Though the microbial diversity increased significantly in the D group than C group, still significant lower than the C group (*p* < 0.01) (Fig. 1a).

Principal coordinates analysis (PCoA) of distances was performed on the relative taxa abundance of the oral microbial communities. We observed a significantly distinct separation in the beta diversity of the oral microbial communities among the C, D and V groups (Fig. 1b).

### The community structure of oral microbiome in all infants

**Fig. 2:**
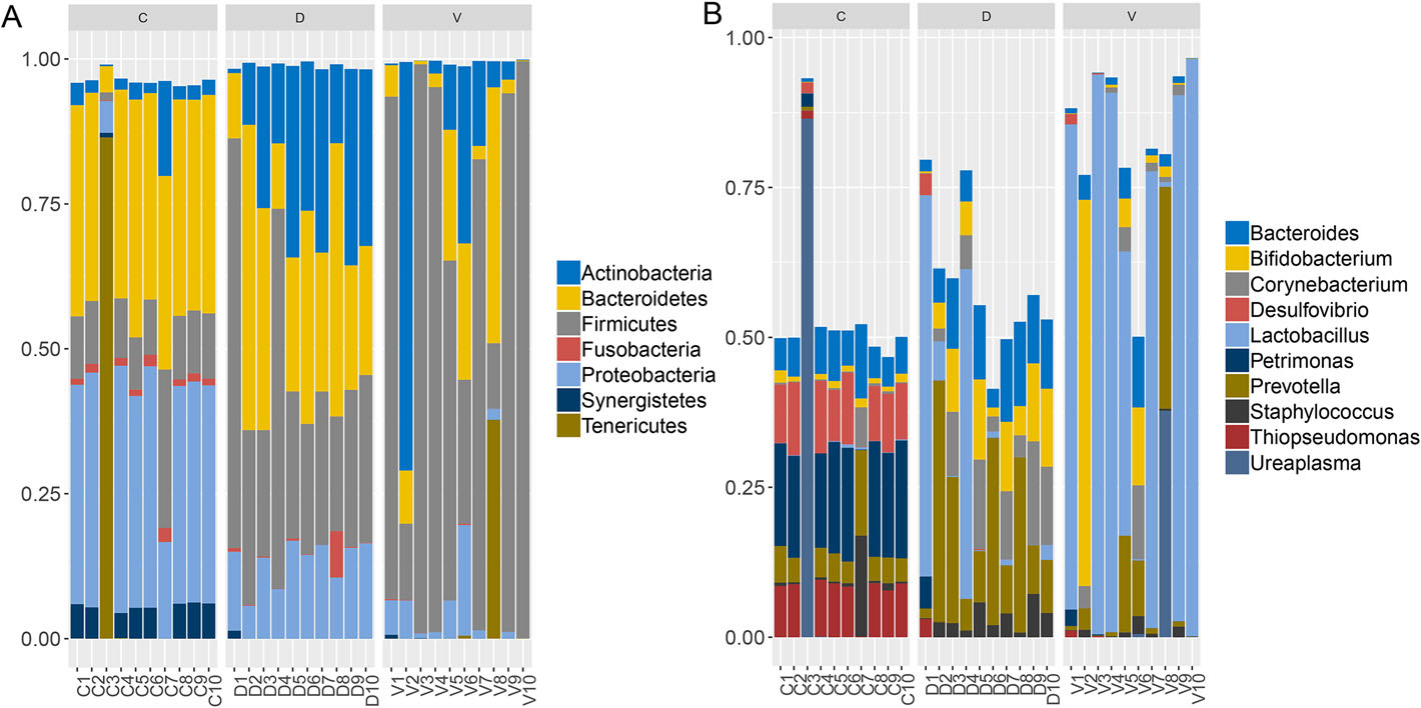
Composition of oral microbiome in infants delivered vaginally with disinfection and without disinfection, and in infants born by cesarean section. **a** Relative abundance at the phylum level. **b** Relative abundance at the genus level

The overall oral microbiota composition of each samples at the phylum level was shown in Fig. 2a. The main phyla were Actinobacteria, Bacteroidetes, Firmicutes, Fusobacteria, Proteobacteria, Synergistetes and Tenericutes. Compared to the V group, the relative abundance of Firmicutes (33.86% versus 66.06%, *p* = 0.02) significantly decreased, while Actinobacteria (21.78% versus 13.70%, *p* = 0.32), Bacteroidetes (28.84% versus 11.26%, *p* = 0.01), Proteobacteria (13.16% versus 4.46%, *p* < 0.01) increased in the D group. In addition, higher abundance of Actinobacteria (21.78% versus 3.60%, *p* < 0.01), Firmicutes (33.86% versus 11.25%, *p* < 0.01), and lower abundance of Bacteroidetes (28.84% versus 33.45%, *p* = 0.42), Proteobacteria (13.16% versus 33.51%, *p* < 0.01) were observed in disinfection group than cesarean section group.

Figure 2b presented the main genera among the enrolled subjects, including Bacteroides, Bifidobacterium, Corynebacterium, Desulfovibrio, Lactobacillus, Petrimonas, Prevotella, Staphylococcus, Thiopseudomonas and Ureaplasma. The most abundant phylum of 7 samples in the non-disinfection group was Lactobacillus*,* with an average abundance of 57.31%. Furthermore, the most abundant genus detected in a single sample (V2) of the non-disinfection group was Bifidobacterium (64.45%). Compared with the non-disinfection group, it was not difficult to find that most of samples in the disinfection group (13.02%) and the cesarean section (0.15%) lacked Lactobacillus.

### Comparison of the genus level of oral microbiome in three groups of infants

**Fig. 3:**
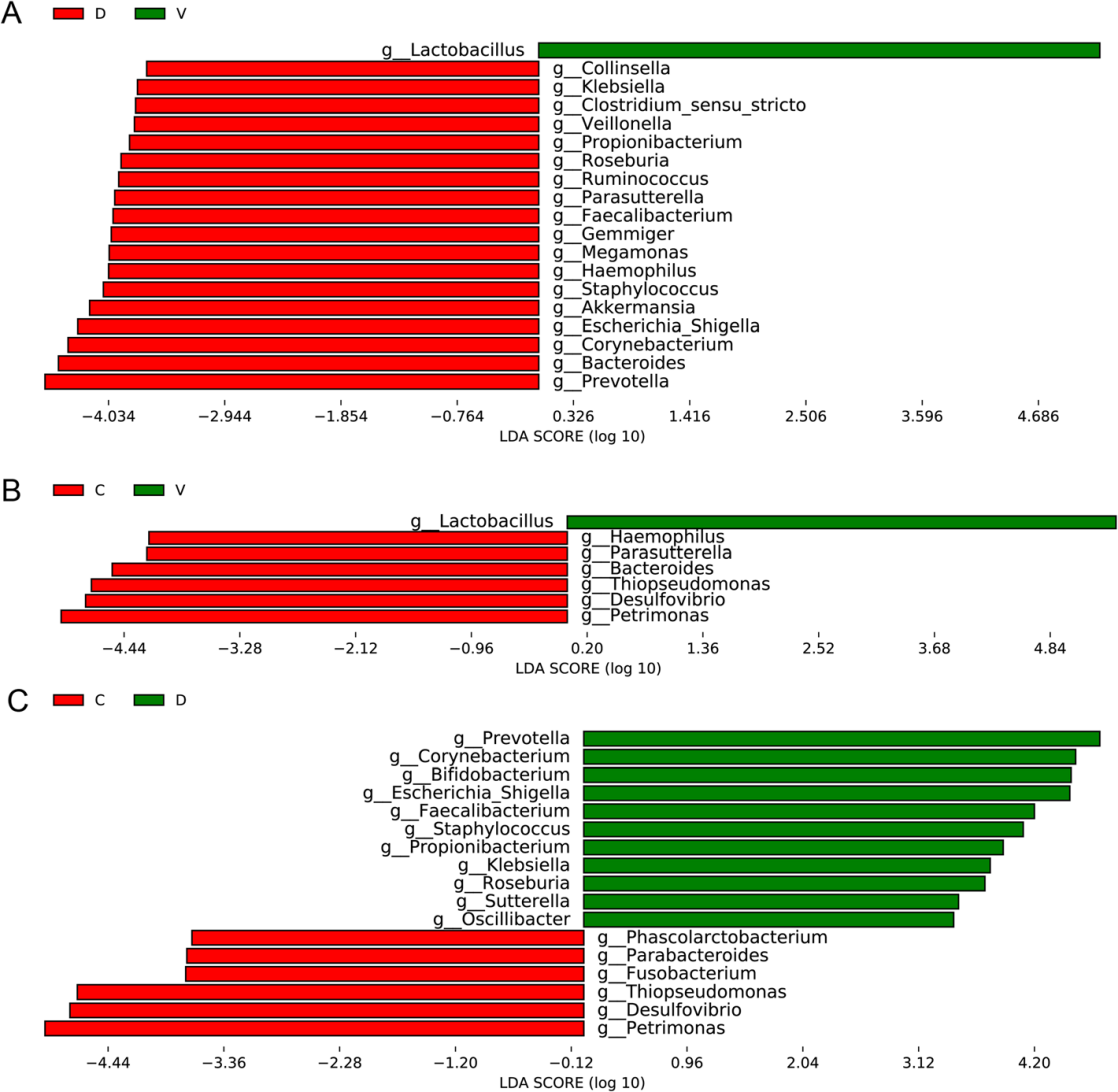
LEfSe comparison of oral microbiome from infants of the three groups. **a** Genus in infants of the non-disinfection group with a positive LDA score were in green, the disinfection group samples with a negative LDA score were shown in red. **b** Genus in infants of the cesarean section group with a negative LDA score were in red, the non-disinfection group samples with a positive LDA score were shown in red. **c** Bacterial genus in infants of the cesarean section group with a positive LDA score were in green, the disinfection group samples with a negative LDA score were shown in red

At the genu level of oral bacteria, we performed a pairwise comparison between group and group with LEfSe software [15]. Through comparing the disinfection group and the non-disinfection group, Fig. 3a clearly showed that Lactobacillus have an obvious increase in the non-disinfection group. Other differential bacteria such as Prevotella, Staphylococcus, Escherichia/Shigella and other opportunistic pathogens showed high relative abundance in the disinfection group. Similar to Fig. 3a, Lactobacillus remained the absolute predominant bacteria in the non-disinfection group compared with the cesarean section group, and other bacteria are relatively reduced (Fig. 3b). Interestingly, we have observed that Lactobacillus has no difference between the disinfection group and the cesarean section. However, there were still more opportunistic pathogens such as Prevotella, Staphylococcus, Escherichia/Shigella in the disinfection group (Fig. 3c).

### KEGG pathway analysis

**Fig. 4:**
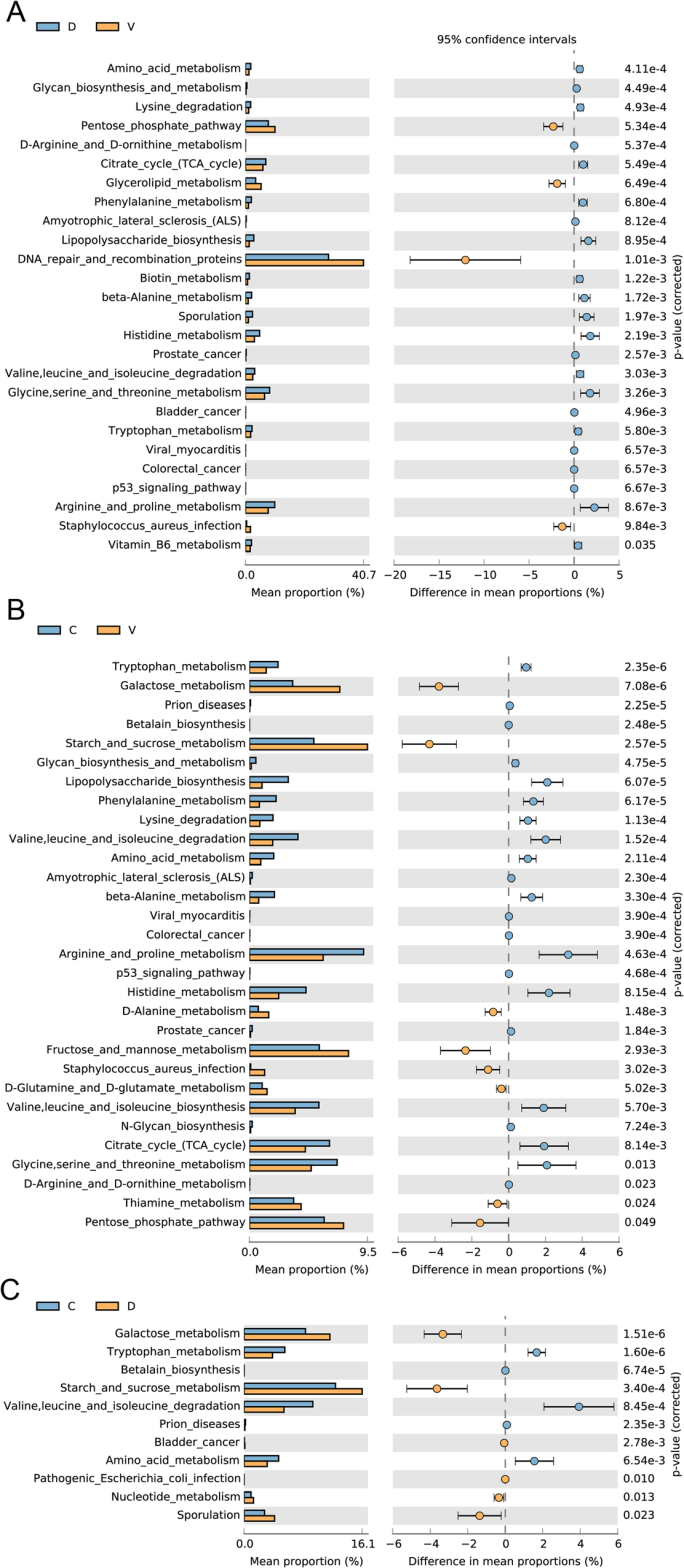
KEGG pathway analysis for oral microflora in three groups. **a** KEGG pathway analysis for oral microflora between the disinfection group and the non-disinfection group. **b** KEGG pathway analysis for oral microflora between the non-disinfection group and the cesarean section group. **c** KEGG pathway analysis for oral microflora between t the disinfection group and the cesarean section group

In order to evaluate the effects of perineal disinfection on the oral microflora of infants, KEGG database was used to compare and predict the metabolic pathways in the oral secretions of the three groups. First, we can see from Fig. 4a, a variety of metabolic pathways significantly increased in the disinfection group, such as amino acid metabolism, arginine and proline metabolism, glycine serine and threonine metabolism compared with the non-disinfection group. Second, we clearly observed that there are still a large number of metabolic pathways in the disinfection group in comparison to cesarean section group (Fig. 4c), such as amino acid metabolism, starch and sucrose metabolism, galactose metabolism. To our astonishment, many harmful pathways (*staphylococcus aureus* infection, viral myocarditis and sporulation) closely related to bacterial infection and bacterial toxins were widely present in the disinfection group (Fig. 4). Moreover, pathways related to prostate cancer, colorectal cancer, amyotrophic lateral sclerosis and p53 signaling pathway all with higher value in the C and D groups (Fig. 4a, b).

## Discussion

A lot of evidence has accumulated showing that the incidence of allergic rhinitis, asthma, eczema and obesity and diabetes in caesarean birth infants is higher than those in vaginal delivery [23, 24]. Changes in intestinal microbiome during early life are the important risk factors for the increased incidence of these diseases [25]. In view of the difference in the mode of delivery, the cesarean section infants can’t inherit the beneficial bacteria from the mothers’ birth canal, so their bacterial structures are different from the infants via vaginal delivery [7, 11, 26]. With the support of the above viewpoint, previous study put gauze in vagina of the pregnant women who need caesarean section, and then smeared infant’ mouth and skin by using the gauze with maternal vaginal secretions after delivery [27]. During follow-up, it was found that these infants’ microbiota was similar to that of vaginal delivery, suggesting that this method or operation could reduce the adverse effects of cesarean section on infants [27]. The textbook “Obstetrics and Gynecology” (Eighth Edition), which is used by Chinese medical colleges and universities, clearly stipulates the requirements of vaginal examination in the chapter of normal delivery to clean the vulva with soapy water first and use polyvidone iodine to disinfect it [13]. In order to understand the implementation of this system, we visited large hospitals and clinics in various regions of China. Unexpectedly found that the execution power of this system reached 100%, the disinfection operation is carried out before the transvaginal examination with povidone iodine in all hospitals and clinics. In the course of clinical practice, on the one hand, the maternity doctors often hold a cotton ball stained with povidone iodine to sterilize the vulva, on the other hand, they directly use a hand impregnated with povidone iodine to enter the mother’s vagina without changing the glove. Because of its large usage and the fact that the parturient is often lithotomy position, povidone iodine is easy to flow into the vagina. With the doubt of whether this medical behavior has an impact on the healthy colonization of the infantile oral microflora, we have done this research hard but perseveringly under the consent of the hospital ethics committee and the maternal and family members.

In this study, the baby’s head was delivered for 1 min without touching the mother’s skin, and samples of oral secretions were collected immediately. Then 16S rRNA sequencing was performed by the second generation sequencing technology detect statistically significant differentially expressed bacteria. Compared with the caesarean section group and the disinfectant group, our experimental results indicated that only Lactobacillus had a high relative abundance in the non-disinfectant group. As the main vaginal bacteria, Lactobacillus plays an important role in maintaining the completeness of intestinal mucosa in healthy women [28]. Moreover, they are recognized as the beneficial bacterium and the precious gift for infant from mother. Compared with the cesarean section group, there was almost no significant difference about Lactobacillus in the disinfection group. Additionally, in contrast to the non-disinfection group and the cesarean section group, increase proportions of potentially harmful bacteria such as Prevotella, Staphylococcus, Klebsiella, Escherichia/Shigella were observed in the disinfection group. However, previous study found that hand alcohol-based disinfectants restricted transmission of opportunistic pathogens [29]. This study was limited to elucidate the mechanism under the increase of these potentially harmful bacteria after disinfection. It should be further investigated with larger sample size and animal and preclinical experiments.

In 2010, the WHO (World Health Organization) survey on maternal and fetal health in Asia showed that the rate of cesarean section in China was as high as 46.2, 11.7% of cesarean section cases without surgical indications have reached the highest level in the world [30]. In recent years, the awareness of maternal and fetal health risks has increased gradually with cesarean section. The hospitals and clinics strictly controlled the cesarean section indexes, and the cesarean section rate has decreased significantly. From the perspective of the establishment of infants’ bacterial microflora, the increasing of beneficial bacteria after vaginal disinfection was no obvious, but the harmful bacteria were increased significantly. In order to estimate the possible hazards, we used the KEGG database to compare the sequencing data of the disinfection group, the non-disinfection group and the cesarean section group. A large number of differential pathways have been investigated, most of which involved amino acid and carbohydrate metabolism. *Staphylococcus aureus* infection, viral myocarditis and sporulation in the disinfection group are involved in bacterial infections and bacterial toxins and are clearly detrimental to the human body. The above evidence suggested that vaginal delivery after disinfection may be more detrimental to the establishment of infant’ oral microflora than the caesarean section delivery, and we are still further tracking the effects on infant’ health and mother’s postpartum recovery. Obstetricians and gynecologists believed that disinfection could reduce wound infections. Whether non-disinfection will lead to wound healing problems requires more clinical data of samples, and we will further advance related research. Another limitation of this study is the missing of information about vaginal bacterial colonization before and after disinfection, which should be taken account in the further study.

## Conclusions

The mode of delivery affects the infant’s Lactobacillus obtained from the mother. Infant with vulvar disinfection not only presented lower Lactobacillus similar to the cesarean section group than the non-disinfection group, but also had more opportunistic pathogens than the cesarean section group. Furthermore, the impact of the above findings on infant health needs further follow-up investigations.

## Data Availability

Sequencing reads and metadata were deposited as entire raw data in the National Center for Biotechnology Information Sequence Read Archive (NCBI SRA BioProject ID PRJNA544373). https://www.ncbi.nlm.nih.gov/bioproject/PRJNA544373/

## Abbreviations

BMI: Body mass index
KEGG: Kyoto Encyclopedia of Genes and Genomes
LEfSe: Linear Discriminant Analysis Effect Size
OTUs: Operational taxonomic units
PCoA: Principal coordinates analysis
PCR: Polymerase chain reaction
PICRUST: Phylogenetic Investigation of Communities by Reconstruction of Unobserved States
rRNA: Ribosomal RNA
WHO: World Health Organization
